# Puberty, brain network connectivity and neuropsychiatric outcomes following pediatric traumatic brain injury in females: A research protocol

**DOI:** 10.1371/journal.pone.0296325

**Published:** 2023-12-29

**Authors:** Abigail Livny, Tamar Silberg

**Affiliations:** 1 Division of Diagnostic Imaging, Sheba Medical Center, Tel-Hashomer, Israel; 2 Department of imaging, Sackler Faculty of Medicine, Tel-Aviv University, Tel-Aviv, Israel; 3 Sagol School of Neuroscience, Tel-Aviv University, Tel-Aviv, Israel; 4 Department of Psychology, Bar-Ilan University, Ramat-Gan, Israel; 5 Department of Pediatric Rehabilitation, Edmond and Lily Safra, Children’s Hospital, Sheba Medical Center, Ramat-Gan, Israel; University of Naples Federico II, ITALY

## Abstract

**Background:**

Examining the role of sex on recovery from pediatric TBI (pTBI) is a complex task, specifically when referring to injuries occurring during critical developmental and maturation periods. The effect of sex hormones on neurological and neuropsychiatric outcomes has been studied among adult TBI females, but not in children. During development, puberty is considered a key milestone accompanied by changes in physical growth, neuronal maturation, sex hormones, and psychological symptoms. Following pTBI, such changes might have a significant effect on brain re-organization and on long-term neuropsychiatric outcomes. While hormonal dysfunction is a common consequence following pTBI, only few studies have systematically evaluated hormonal changes following pTBI.

**Aims:**

To describe a multimodal protocol aimed to examine the effect of puberty on brain connectivity and long-term neuropsychiatric outcomes following TBI in female girls and adolescents.

**Methods:**

A case-control longitudinal prospective design will be used. 120 female participants aged 9 to 16 years (N = 60 per group) will be recruited. In the acute phase (T0-1 month), participants will undergo an MRI protocol for brain connectivity, as well as a clinical evaluation for puberty stage and hormonal levels. In the chronic phase (T1-18-24 months), participants will complete a neuropsychiatric assessment in addition to the MRI and puberty evaluations. Hormonal levels will be monitored at T0 and T1. A moderation-mediation model will be used to examine the moderating effects of puberty on the association between pTBI and neuropsychiatric symptoms in female girls and adolescents, through the mediating effect of brain network connectivity.

**Significance:**

This study will highlight sex-specific factors related to outcomes among females following pTBI and enhance our understanding of the unique challenges they face. Such information has a substantial potential to guide future directions for research, policy and practice.

## Introduction

In the last decade, policy statements have been made by funding agencies regarding the requirements for the consideration of sex and gender [1] in research, emphasizing the fundamental contribution of these factors for good scientific practice [1, 2] (in our study we will refer to sex as the endogenous variable, although we acknowledge that sex and gender constructs are interconnected and not binary). The World Health Organization (WHO) Disability and Rehabilitation Action Plan 2014–2021, also calls for including age and sex data in policy documents among individuals with developmental and acquired disabilities [3], such as brain injury. Traumatic brain injury (TBI) is considered a silent epidemic, as the major post-TBI disabilities, predominantly neuropsychiatric, are not immediately apparent. Furthermore, sex effects on the pathophysiology and recovery are poorly understood. In recent years there is a growing recognition of a *male bias* problem, with predominantly rodent males used in preclinical TBI designs and fewer females than males recruited to clinical trials. Without research, which focuses specifically on the mechanisms and outcome of females with TBI there is a risk that clinical care, policy development and advocacy services will not effectively accommodate post-TBI females.

Outcomes following TBI show that females experienced worse outcomes beyond injury severity [4], took longer time to recover [5], showed higher post-concussive symptoms [6], greater cognitive decline [7], and a higher risk of depression compared to males [8, 9]. In contrast, a recent review found better quality of life [10] and better cognitive functioning [11] among females following TBI, as compared to males. Such inconsistencies in the literature require an in-depth evaluation of additional factors that may affect recovery and outcome post TBI focusing not only on sex-differences, but rather on sex-specific factors.

Sex hormones, (specifically gonadal hormones such as estrogen and progesterone) have been suggested as significant factors mediating neurological and neuropsychiatric outcomes after TBI in females [12, 13]. A possible explanation, known as the *hormonal withdrawal hypothesis* (i.,e, frequent miss of menstrual periods or total absence of menstruation), has been reported in females following TBI [13], and suggested to be a key factor contributing to worse outcomes in females. Furthermore, as males have low pre-injury levels of gonadal hormones, they may be less affected by hormonal suppression post injury. The phase of the menstrual cycle at injury, was found to be significantly related to psychological and neurologic outcomes among females following mild TBI [13]. Females with the highest progesterone levels at the time of injury (i.e., luteal phase of the menstrual cycle) had the worst outcomes as compared to females in the follicular phase (i.e., relatively low progesterone levels) and compared to females taking synthetic progestin (i.e., maintaining high levels of progestin before and after injury). Thus, if the hormonal withdrawal hypothesis is linked to poorest outcomes, a relatively simple intervention could lead to significant improvement in outcomes [13] necessitating the need to address hormonal levels and time of menstrual cycle across studies with female TBI patients of all ages.

While there is paucity of data on the effect of sex on neurological and psychological outcomes in adult TBI females, there is even sparser literature on such sex effects among female girls and adolescents. Pediatric-TBI (pTBI), a leading cause of disability in children and adolescents [14] can lead to physical, neurological, cognitive and neuropsychiatric disorders [15, 16]. Many factors have been shown to affect outcomes of pTBI, among them are: pre and post-injury environmental factors [17], injury severity [18], time since injury [19, 20], and child’s sex and age at injury [15, 21, 22]. During development, age may mask differences in outcomes related to the role of sex on recovery, specifically when referring to injuries occurring during critical developmental and maturation periods. Emerging literature suggests that both the response to injury and the path to recovery may differ between male and female children and adolescents [21, 23]. Unfortunately, research on pTBI seldom separates females and males in their analyses [24], and the literature on outcomes is relatively limited. A recent study revealed that pTBI patients had a 3.2-fold higher odds of being diagnosed with an endocrine disorder, with even higher rates in females compared to males [25]. Both early and late endocrine changes can occur after pTBI including acute alterations in the hypothalamic-pituitary-adrenal (HPA) axis, and disturbances in puberty [26, 27]. In addition, an interaction between sex, and neuropsychiatric symptoms following pTBI was reported, with males affected more than females [28], and females being at a higher risk for developing internalizing symptoms (e.g., depression and anxiety) [29], while males tend to present more externalizing problems (e.g., impulsive and aggressive behaviors) [30].

Hormonal dysfunction following pTBI is common and can evolve, resolve, or persist after injury [31]. It is not clear how much of the puberty related hormonal changes contribute to variation in pTBI outcomes [31]. Puberty is a key developmental milestone accompanied by substantial physical growth, neurologic changes, peak levels of sex hormones, and changes in emotional and behavioral symptoms [32]. It encompasses a combination of two distinct brain activated hormonal processes, adrenarche (the activation of the HPA axis) and gonadarche (the reactivation of the hypothalamic-pituitary-gonadal axis), which together lead to a dramatic rise in the circulating levels of sex hormones [33]. Puberty is associated not only with an increase in hormone levels, but also with dramatic fluctuations in the levels of estrogen circulating in the body [34]. Subsequently, the body’s systems must rapidly adapt to higher and fluctuating levels of hormones that may influence mood and other emotional symptoms. Recently, a large cohort study [35] demonstrated that earlier onset of puberty (pubescence) was associated with higher risk of depression and anxiety in females but was unrelated to the presence of depression in males. The gap between the onset of emotional changes and full maturation of inhibitory brain mechanisms may contribute to a window of risk for emotional dysregulation and other neuropsychiatric symptoms during puberty [36]. Paucity of studies have indicated that pubertal status predicts sex difference in the prevalence of neuropsychiatric symptoms better than age, suggesting that sex hormones play a significant role in the pathophysiology of neuropsychiatric disorders [34]. Furthermore, it should be acknowledged that age and pubertal maturation are highly correlated, arising the need for research designs that explicitly disentangle puberty and age effects on pTBI outcomes [37]. This requires the comparison between females with and without pTBI within the same age range and puberty phase.

Studies of pTBI survivors have reported high prevalence of pituitary dysfunction related to hormonal deficiency (hypopituitarism) at 1–5 years post injury [38]. Interestingly, injury severity did not predict endocrine outcomes. In a recent study, p-TBI patients had about 3-times the risk of a central endocrine diagnosis compared to the general population. Moreover, females had a shorter time gap between the injury and the endocrine disorder compared to males [25]. Probable pubertal adolescent females (13–18 years) had a reduced mortality rate compared to males. These findings suggest that post-injury elevation in hormonal levels among young females may propose an opposite profile to the withdrawal hypothesis reported in adult TBI females.

The relationship between puberty and brain development is likely to be complex and nonlinear, and interacting with the distinct but synchronous effects of age [33]. There is evidence for brain structural development associated with pubertal maturation [39, 40] with different trajectories seen in males and in females [39, 41]. Evidence of a strong relationship between white-matter fiber density and age, sex, and puberty during development has been reported [42, 43]. Physical pubertal changes predicted patterns of changes in structural connectivity, and pubertal processes contributed to distinct changes in males and females across brain development [44]. Studies using structural brain network graph theory revealed an early separation between developmental trajectories of males and females, with male adolescents displaying higher structural intra-hemispheric connectivity and females displaying higher inter-hemispheric connectivity [45]. Although studies on brain connectivity and pubertal hormones are limited, there is evolving evidence for the hypothesis that sex-hormones are related to the microstructure of white-matter and that pubertal development may be associated with maturation of structural connectivity above and beyond chronological age [46].

**Fig 1:**
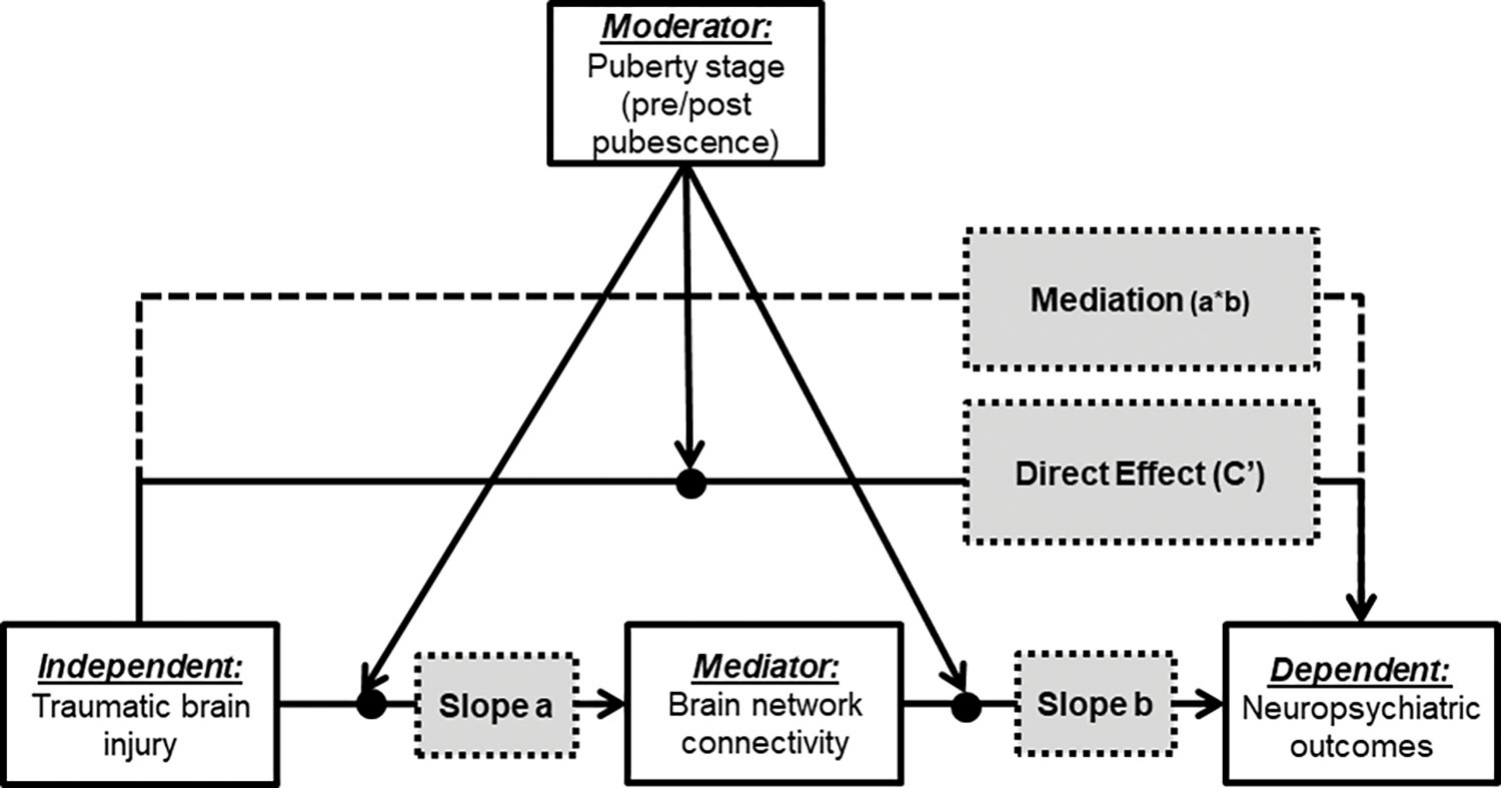
The study’s moderated-mediation model: The association between pTBI and neuropsychiatric symptoms (direct effect (c’)) as mediated by brain network connectivity (mediation (a*b)), and moderated by puberty stage.

Taken together, the current research question forms a moderated-mediation model using a longitudinal case-control design. The main aim of the proposed study is to examine the moderating effect of puberty (pre-and post-pubescence) in females, on the association between brain network connectivity and neuropsychiatric outcomes in girls with/without pTBI. Fig 1 illustrates the study’s conceptual model.

### Aims and hypotheses

Based on the literature review, the present study proposes the following aims and hypotheses (as depicted in Fig 1):

**Aim 1**: Examine the moderating effect of puberty (pre- and post-pubescence) on the association between pTBI, brain network connectivity measures and neuropsychiatric outcome among females:

1.1. Examine the direct association between pTBI and neuropsychiatric outcome (Fig 1: direct effect c’ pathway). We hypothesize that females following pTBI will present poorer neuropsychiatric outcome as compared to healthy female controls.

1.2. Examine the relationship between pTBI and brain network connectivity measures (Fig 1: slope a pathway). We hypothesize that females with pTBI will present higher network connectivity measures and lower network efficiency compared to healthy female controls.

1.3. Examine the relationship between brain network connectivity measures and neuropsychiatric outcome (Fig 1: slope b pathway). We hypothesize that higher network connectivity measures and lower network efficiency will be associated with poorer neuropsychiatric outcome.

1.4. Examine the moderating effect of puberty on the mediated association between pTBI, brain network connectivity and neuropsychiatric outcome (Fig 1: mediation a*b pathway through moderator). We hypothesize that puberty will moderate the associations between brain network connectivity measures and network efficiency, and neuropsychiatric outcome in females post pTBI. That is, post-pubescence females compared to pre-pubescence females will show higher network connectivity measures, lower network efficiency and poorer neuropsychiatric outcome.

**Aim 2:** Examine the effect of changes in puberty stage and hormonal levels on brain network connectivity measures and neuropsychiatric outcome in pTBI females. We hypothesize that among females following pTBI, earlier changes in puberty stages (i.e. changes from one Tanner stage to another) and a significant increase in hormonal levels across recovery, will be related to higher network connectivity measures and lower network efficiency as well as to poorer neuropsychiatric outcome.

## Methods

The proposed study is a part of a larger study, looking at brain and neurocognitive outcomes among male and female children and youth with various acquired brain injuries approved by both Sheba Medical Center (#3211-16-SMC) and Ministry of Health ethics (#20162005) committees, and was registered at the National Institute of Health (*NIH#*: *NCT03034031*). The unique data to be especially collected for this study includes sensitive puberty measures enabling a thorough examination of their effect on brain and neuropsychiatric outcomes and was preregistered in Open Science Framework (https://doi.org/10.17605/OSF.IO/FZWCE, version #1, 08/22). Data will be deidentified, saved with password and stored in a secured Hospital Server, with access limited to study research team. Trained clinic staff will describe the purpose of obtaining blood samples and MRI images, steps taken to protect the participant’s confidentiality and privacy in terms of how images and records would be managed and how research would be presented, a description of the MRI procedure, and a discussion of how participant safety would be maintained. All participants will enter voluntarily, their parents will sign a written informed consent form and the female participants will sign a written assent form indicating their understanding and acknowledgment of study’s procedures. The form will include an approval to share de-identified clinical data in adherence with local Institutional Review Board and other local ethical policies aimed at protecting patient privacy.

### Research design

The proposed study will use a longitudinal prospective design and follow females after pTBI and age matched healthy female controls, aged 9 to 16 years [47]. Participants will be followed from admission to 18 months post injury. Participants will undergo an MRI protocol, a short neuropsychiatric assessment and a clinical assessment of puberty stage and hormonal level, across two time points: **Acute (T0)**: within one month post injury; **Chronic (T1)**: 18 months from T0 (sufficient time for neuropsychiatric changes post pTBI [48]). For a description of the schedule of study’s enrolment and assessment periods see S1 Table.

### Sample size and power

Sample size was calculated on the basis of our primary hypothesis that puberty will moderate the associations between brain network connectivity measures, and neuropsychiatric outcome in females post pTBI. The bootstrap method for moderated-mediation models was used [49]. The bootstrap method, based on resampling, is useful for finding the standard errors and forming percentile-based confidence intervals (CIs) for estimates when their sampling distributions are unknown. According to Preacher, Rucker & Hayes (2007), a minimum of 100 participants are required in order to achieve power of at least .80, at a significance level of α = .05, with a Cohen’s d medium effect size (corresponding to a value of 13% of the variance in predicted variable [50]; and in accordance with previous studies on neural correlates of neuropsychiatric outcomes in children with TBI [51]). Based on the literature on longitudinal studies with pTBI patients, and due to technical issues such as unusable scans due to excessive movement anticipated in a pediatric study, we anticipate an attrition rate of ≈20% [52]. Thus, 120 female participants will be recruited: 60 with pTBI and 60 healthy controls.

### Measures of study variables

#### 1. Independent variable

*Clinical TBI group*. Participants will include females following mild to moderate-severe injuries. Patients will be recruited from the Pediatric Emergency Department, which treats ~200 children diagnosed with mild- TBI annually with ~30% females, and from Pediatric Rehabilitation Departments (PRD) of both Edmond and Lily Safra Children’s Hospital, Sheba Medical Center and Loewenstein Hospital, Israel. Each department treats ~35 pTBI patients annually with ~30% females.

Injury severity will be defined based on a composite index according to child’s Glasgow Coma Scale (GCS), Duration of Post Traumatic Amnesia (PTA) and duration of Loss of Consciousness. As no single measure of injury severity has been found to be reliable among children, using an index score will allow us to define injury severity as a continuous variable ranging from complicated-mild to severe with greater accuracy.

*Healthy control group*. Healthy age matched female participants will be recruited from the general population or from siblings of pTBI females.

Inclusion criteria: 1. for pTBI: All injury severities; 2. For both pTBI and healthy controls: Females aged 9–16 years (based on the 95%CI of age range sensitive for attaining pubertal milestones) [47]. Exclusion criteria for both groups: 1. History of neurological disorders, intellectual disorders, developmental disorders or previous head injury; 2. Cardiovascular instability; 3. Fever or evidence of microbiological contamination; 4. Uncontrolled seizures; 5. MRI exclusion criteria, such as metal implants. 6. Endocrinology exclusion parameters: known abnormality of gonadotrophin secretion or of ovarian function. In addition, all MRI scans will undergo a neuroradiological examination, and abnormal findings (aside from their brain injury for the pTBI group), will be reported to the Institutional Ethics Committee as well as to the participant. Following this, participant’s participation in the study will be stopped.

### Mediator

#### Changes in network brain connectivity

A score indicating differences in brain network connectivity measures between T1 and T0 will be defined and used as the mediating variable in all analyses. Children will be scanned on a 3T Magnetom Prisma (Siemens Healthineers) MRI system. Structural scans, T1-weighted, fluid attenuation inversion recovery (FLAIR) and susceptibility-weighted imaging (SWI) will be acquired for clinical neuroradiology report. During scans, participants will watch animated videos, in order to achieve better compliance in the scanner.

*Structural connectivity*. Will be measured using a diffusion weighted imaging scan. Spin-echo diffusion-weighted, echo-planar imaging sequence will be performed with 76 axial slices, voxel size 2x2x2mm, FOV = 240x240, matrix = 120x120, TR = 4500ms, TE = 51ms, MB = 2; b-value = 1,000 s/mm^2^, 64 gradient directions, 3-b0 images. Regularization, brain extraction, and motion correction will be conducted. Diffusion tensors will be calculated using a nonlinear regression procedure [53]. Whole brain tractography will be performed based on deterministic streamline fiber tractography approach [54].

*Brain network architecture of structural connectivity*. Using Brain connectivity toolbox, structural connectivity matrices will be calculated, by which, nodes indicating anatomical elements (brain regions) and edges, representing the relationships between nodes (white matter tracts), will be defined. These connectivity matrices will be further used for graph theory analyses, allowing to quantitatively characterize the global organization via network measures [55]: (1) **centrality**: strength (number of fibers connected to each node) and betweenness centrality (number of shortest paths passing through the target node); (2) **integration**: global efficiency (the shorter the path between two regions, the more efficient the connection), and (3) **segregation**: local efficiency and cluster coefficient: (reflects the tendency of nodes to cluster together). Lesions will be classified using a scheme of Adams et al. (1989) [56], to identify 3 diffuse axonal injury grades. We will further assess the relationships between network measures and the lesion grading score [57].

#### 2. Moderator

*Pubertal stage*. Pubertal stage will be assessed at injury/first visit for controls (T0), and at 18 months from T0 (T1). Pubertal stage will be defined based on 3 different measures, known to be highly correlated [58]: (1) the Pubertal Development Scale (PDS) [59]. This self-report questionnaire contains questions concerning secondary sexual characteristics, including items concerning hair growth, skin changes, and growth spurt, with sex-specific items (i.e. menarche and breast development in females). The PDS measures a composite puberty score including effects of adrenal, and gonadal hormones, as well as growth hormone. Participants will be instructed to indicate pubertal stage on each of these physical characteristics on a 4-point scale: ranging from (a) has not started to develop, (b) shows first signs of development, (c) shows clear development to (d) has finished developing; (2) *Hormonal Levels* (LH, FSH and Estradiol) will be drawn from blood samples at T0 and T1. For females who are post menarche, and T1 assessment meetings will be scheduled at 3–5 days of the menstrual cycle; and (3) *Tanner Stage* assessment according to a physical examination conducted by a qualified clinician. Tanner staging categorizes individuals along an ordinal puberty scale from 1 to 5, on the basis of pubic hair and breast development in females [60]. The majority of girls reach Tanner Stage II at age 10.5 (a proxy for pubertal onset), and then progress through an average rate of 0.96 stages per year [61]. A binary puberty (pre and post-pubescence) score at T0 will be computed out of the pubertal stage measures and used as the moderator variable in the statistical model. If the different measures will conflict (i.e., yield different puberty stage classifications), the final score will be based on the Tanner stage score, which will be further used in analyses [47].

#### 3. Dependent variable

*Neuropsychiatric symptoms*. Assessment of neuropsychiatric outcomes will comprise three different measures including: objective cognitive task, parental proxy report and participant’s self-report.

1. A cognitive task (Go/No-Go) will examine response inhibition (RI), a central component of executive control [62], which is considered a sensitive measure for underlying risk for neuropsychiatric symptoms [63].

2. Proxy report—parents will complete the Child Behavior Checklist (CBCL) from the Achenbach System of Empirically Based Assessment (ASEBA) [64], which is considered a “gold standard” tool for assessing psychopathology among children and adults and has been used among pTBI patients [20, 23, 65].

3. Self-report–Emotion regulation questionnaire for children/adolescents (ERQ-CA) [66] and the response to positive affect scale for children (RPA-C) [67] will be used to examine participants’ perception of their own emotional regulation skills. These two forms of emotion regulation depend upon interactions between prefrontal and cingulate control systems, cortical and subcortical emotion generative systems [68], and are associated with neuropsychiatric symptoms [69].

#### 4. Covariates

*Pre-injury factors*. Demographic information regarding family’s socioeconomic status (SES), maternal educational level, ethnicity and religiosity will be collected at T0. Since Parents’ mental health pre and/or post injury may also affect a child’s neuropsychiatric status [70, 71], the General Health Questionnaire (GHQ-12) [72] will be used as a proxy of parents’ mental health. Parent’s will complete the CBCL at admission to reflect pre-injury functioning.

### Analytical approach

The following analyses will be conducted on both pTBI patients and healthy controls:

#### Descriptive statistics

Descriptive statistics of variables will be obtained to understand the characteristics of the study population, to evaluate the distribution of participants across subgroups (e.g., puberty stage), and to identify potential confounders to inform subsequent analyses.

**Aim 1**- **moderated-mediation model:** To test our main study’s hypothesis we will apply a moderated- mediation model. Briefly, we will examine whether the magnitude of a mediation effect impact of mediator (*changes in brain network connectivity*) on the association between predictor (*pTBI*) and outcome (*neuropsychiatric outcomes*) is conditional on the value of a moderator (*puberty stage at T0*). The analysis of the moderated-mediation model will use Hayes’s (2013) PROCESS macro (Model 59) [73]. Bootstrapping method will test significance of the effects to obtain robust standard errors for parameter estimation [73]. This will produce 95% bias-corrected confidence intervals of these effects from 1000 resamples of the data. Confidence intervals that do not contain zero indicate significant effects at *p* < .05.

**Aim 2- effect of changes in puberty stage and hormonal levels on mediator and outcome:** Two step-wise linear regression models controlling for age and pre/post-injury factors in the 1st step; for the injury severity in the 2nd step; and for puberty stage and hormonal levels at T0 and T1, as well as the difference between both measures (i.e., *Δpuberty stages and Δhormonal levels*) in the 3^rd^ step, to predict (1) brain network connectivity and (2) neuropsychiatric outcomes, at T1 will be conducted.

#### Exploratory analyses

While our primary moderated-mediation model will interrogate each pathway separately, in supporting analysis we will apply structural equation models (SEMs) to further examine the hypotheses linking pTBI and brain changes to neuropsychiatric outcome via multiple puberty measures measured at different time phases, in a single modeling framework. In case of partial mediation, this approach also allows for simultaneous estimation of direct and indirect effects of pTBI and puberty stage on connectivity changes and neuropsychiatric outcomes. Here, the model structure will be informed by findings from primary analyses, and overall fitness of the model will be assessed comprehensively using standard fit indices [74]. Upon establishing goodness of fit, standardized coefficients for each pathway will be used to estimate both direct and indirect effects.

#### Secondary analyses

For all analyses, an equal distribution assumption of potential confounders (participants’ age, injury severity, time since injury, and pre/post-injury factors) will be tested and models augmented with terms for these confounders will be considered.

Puberty stage: in our primary analyses, we will use a binary puberty score (pre/post pubescence). Secondary analyses will consider each of the puberty measures (PDS, hormonal levels, Tanner stage) separately.

Neuropsychiatric outcomes: our primary analyses will use a composite global neuropsychiatric risk score based on the ASEBA Summary scales as our main outcome. In secondary analyses, we will consider each of the neuropsychiatric measures (IR, ASEBA, Emotional Regulation) separately.

### Preliminary studies supporting the proposed aims

To support the proposed study, we show results of both sex and puberty effects on neuropsychiatric outcome in pTBI patients. Our preliminary data highlight the potential impact of the proposed study to identify the moderating effect of puberty on the association between pTBI, brain connectivity and neuropsychiatric symptoms.

**Fig 2:**
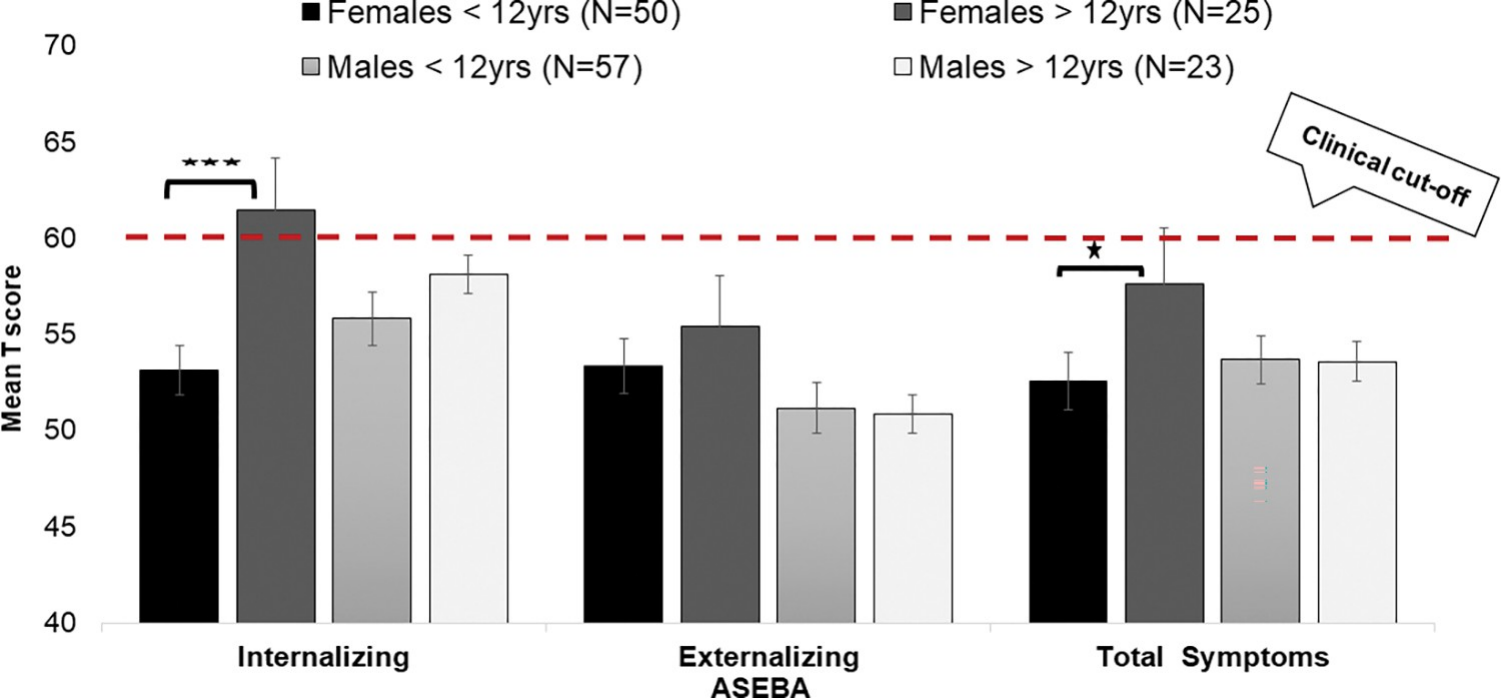
Age and sex effects on neuropsychiatric symptoms among females and males following pTBI.

To support our main aim regarding the effect of puberty on long term neuropsychiatric outcomes following pTBI (aim 1 and 4), a retrospective analysis among 155 TBI children and youth (48% females) hospitalized at the PRD between 2010–2016 following was conducted. The sample was divided into 4 (2x2) groups according to sex [female vs male] and age [below and above 12 years], with the 12 years cutoff serving as a proxy for pubescence [75, 76]. Long-term (Mean = 20 months post injury) neuropsychiatric symptoms were evaluated using the ASEBA questionnaires. A sex by age effect was found (Fig 2) with females in the >12 years group presenting higher rates of total-problems scores [*F*(1,74) = 4.97, *p* = 0.03, d = 0.2] compared to females in the <12 years group. No significant differences were found between males in the below and above 12 years groups. These results suggest that female sex and puberty level (in the pilot study according to age^86^) serve as risk factors for neuropsychiatric symptoms post pTBI.

To support aim 1 further analysis was conducted in relation to the levels of clinical symptoms in our female sample as compared to norms. We found that the median scale score for Internalizing symptoms among females in the > 12 years age group was T = 63, which is above the clinical-band cutoff of the scale of the questionnaire (T = 60), indicating that approximately 50% of the female adolescents (>12 years) following TBI experienced clinical levels of anxiety and depression. Such percentage is extremely high compared to the percentage of internalizing symptoms in the general population (odds-ratio of 50/3 [OR = 16.7]).

To support aim 2 and aim 3 graph theory measures of structural connectivity were calculated among N = 5 females following pTBI and N = 1 healthy girl in a longitudinal design. We found global decreases in adult chronic TBI patients, in structural graph theory measures of strength, efficiency and cluster coefficient, suggesting that TBI causes an extensive disruption of integration, segregation and centrality within the brain network [77]. In the pediatric feasibility sample N = 3 were pre-menarche (age: 10.67(1.15) years; GCS:14.67(0.58); PTA<1 day; LoC< 30 min) and N = 2 were post-menarche (age: 16.5(3.53) years; GCS:7(1.41); PTA<7 day; LoC< 24 hours). Pre-menarche females following pTBI presented lower values of brain network connectivity measures compared to post-menarche patients. Moreover, females following pTBI presented a decrease in all network measures from acute to chronic phase. Finally, pre-menarche pTBI patients exhibited lower values of strength and efficiency than the healthy control who was lower than the post-menarche pTBI patients (Table 1). *Overall*, *the above preliminary results* highlight the need to specifically address puberty levels and hormones among females following pTBI to better identify their moderating effect on brain connectivity and neuropsychiatric symptoms, to inform on the design of future clinical trials.

**Table 1:** Graph theory measures of females pre- and post-menarche across two time-points.

Group	Measures	Time 1 Mean±SD	Time 2 Mean±SD
**Pre-menarche pTBI**	Strength	19.52(2.18)	18.78(0.11)
	Efficiency	84.86(9.55)	83.36(2.96)
	Clustering	0.038(0.004)	0.031(0.009)
**Post-menarche pTBI**	Strength	23.91(0.82)	22.61(1.01)
	Efficiency	111.20(20.17)	93.04(10.22)
	Clustering	0.036(0.004)	0/032(0.004)
**Pre-menarche Healthy controls**	Strength	20.59()	-
	Efficiency	91.83()	-
	Clustering	0.032()	-

The impact of the proposed research are three-fold: (1) on a theoretical level, it will characterize puberty related pathophysiological changes in females occurring during recovery from pTBI, and enable prediction of neuropsychiatric outcomes; (2) on a methodological level, employing brain network graphical connectomics related to puberty changes, together with neuropsychiatric evaluations, may have important implications for the development of new neuroimaging protocols for the assessment of brain re-organization among pTBI females; and (3) on a clinical level, a multidisciplinary approach recognizing the effect of puberty on the developing brain and understanding recovery mechanisms may provide biomarkers to aid in developing treatment protocols and recommend on specific guidelines for clinical monitoring of pTBI females.

### Dissemination plan

Publication methods will include press releases, annual reports, presenting at national and international scientific conferences events (e.g. Human Brain Mapping, International Neuropsychology Society, Neurotrauma, American Academy of Child and Adolescents Psychiatry), and authors will submit manuscripts for publication in peer reviewed journals in the field of neuroimaging, child neuropsychology and neuropsychiatry. Furthermore, the authors plan to work with stakeholder groups to interpret the project learnings in order to move forward a larger multi-site research study to build a significant evidence base for the current work. Within the next study building clinical capacity at additional local and international sites will be developed. Such work can serve as the basis for the creation for clinical recommendations in the field of childhood neuropsychiatry.

## Supporting information

S1 Table Schedule of study enrolment and assessment periods. (TIF)
