# Supplementary figures and images for: Puberty, brain network connectivity and neuropsychiatric outcomes following pediatric traumatic brain injury in females: A research protocol
Source: PLoS One. 2023 Dec 29;18(12):e0296325. doi: 10.1371/journal.pone.0296325 (PMC10756517; doi:10.1371/journal.pone.0296325)

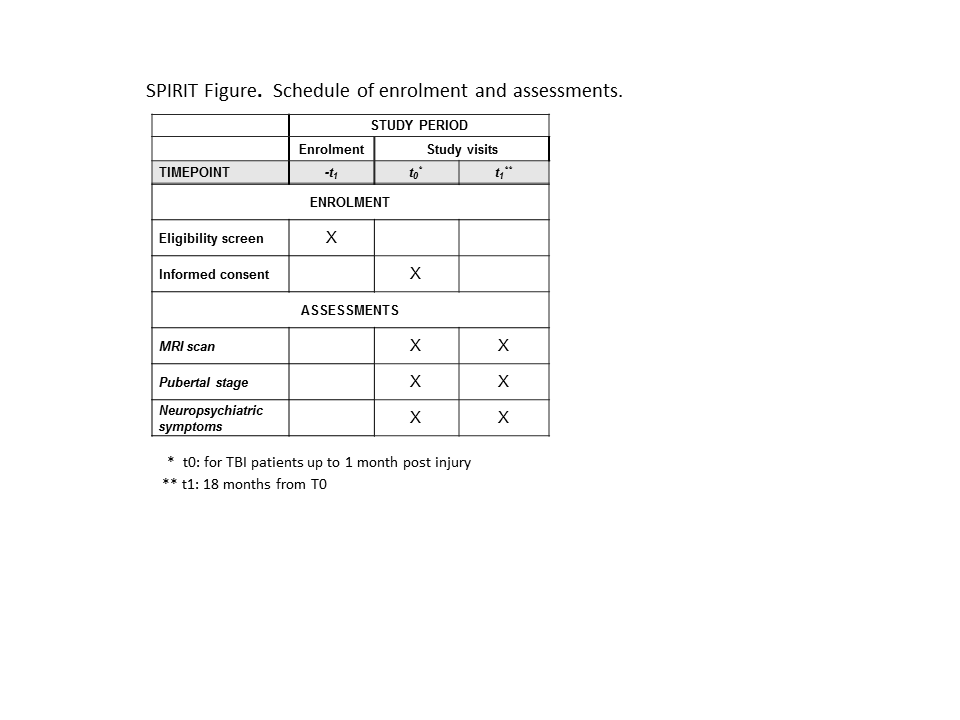

Supplement: S1 Table — (TIF) [file pone.0296325.s001.tif]
